# Leaf and Root Endospheres Harbor Lower Fungal Diversity and Less Complex Fungal Co-occurrence Patterns Than Rhizosphere

**DOI:** 10.3389/fmicb.2019.01015

**Published:** 2019-05-08

**Authors:** Xin Qian, Hanzhou Li, Yonglong Wang, Binwei Wu, Mingsong Wu, Liang Chen, Xingchun Li, Ying Zhang, Xiangping Wang, Miaomiao Shi, Yong Zheng, Liangdong Guo, Dianxiang Zhang

**Affiliations:** ^1^Key Laboratory of Plant Resources Conservation and Sustainable Utilization, South China Botanical Garden, Chinese Academy of Sciences, Guangzhou, China; ^2^College of Life Sciences, University of Chinese Academy of Sciences, Beijing, China; ^3^State Key Laboratory of Mycology, Institute of Microbiology, Chinese Academy of Sciences, Beijing, China; ^4^Biomarker Technologies Corporation, Beijing, China; ^5^Key Laboratory of Pathogenic Microbiology and Immunology, Institute of Microbiology, Chinese Academy of Sciences, Beijing, China; ^6^Key Laboratory of Humid Subtropical Eco-geographical Process of Ministry of Education, Fujian Normal University, Fuzhou, China

**Keywords:** fungal community, fungal endophyte, *Mussaenda kwangtungensis*, high-throughput sequencing, niche differentiation

## Abstract

Plant-associated microbiomes are key determinants of host-plant fitness, productivity, and function. However, compared to bacterial community, we still lack fundamental knowledge concerning the variation in the fungal microbiome at the plant niche level. In this study, we quantified the fungal communities in the rhizosphere soil, as well as leaf and root endosphere compartments of a subtropical island shrub, *Mussaenda kwangtungensis*, using high-throughput DNA sequencing. We found that fungal microbiomes varied significantly across different plant compartments. Rhizosphere soil exhibited the highest level of fungal diversity, whereas the lowest level was found in the leaf endosphere. Further, the fungal communities inhabiting the root endosphere shared a greater proportion of fungal operational taxonomic units (OTUs) with rhizosphere communities than with leaf fungal endophyte communities, despite significant separation in community structure between the two belowground compartments. The fungal co-occurrence networks in the three compartments of *M. kwangtungensis* showed scale-free features and non-random co-occurrence patterns and matched the topological properties of small-world and evidently modular structure. Additionally, the rhizosphere network was more complex and showed higher centrality and connectedness than the leaf and root endosphere networks. Overall, our findings provide comprehensive insights into the structural variability, niche differentiation, and co-occurrence patterns in the plant associated fungal microbiome.

## Introduction

Plant associated fungal communities exert profound and crucial influence on plant survival, health, productivity and even ecosystem functions (Berg et al., 2005; Rodriguez et al., 2009). Therefore, plants may be viewed not as standalone entities but rather as holobionts consisting of the hosts and their associated mycobiome that usually referred to as the host’s second genome, which are together cohesive evolutionary units of selection and biological organization (Zilber-Rosenberg and Rosenberg, 2008; Bordenstein and Theis, 2015; Fonseca-García et al., 2016; Beckers et al., 2017). A better understanding of the community assembly and ecological interactions of plant associated fungi could improve our ability to manipulate these microorganisms to practical application, such as increasing agricultural productivity, phytoremediation and providing antimicrobial compounds (Waller et al., 2005; Tejesvi et al., 2013). Although the plant holobiome is gaining increased attention, little work has been conducted to explore the niche differentiation in their community structure and co-occurrence patterns associated with the leaf endosphere, root endosphere, and the rhizosphere compartments.

Endophytic fungi are functionally dominant members of plant microbiome; they are capable of occupying host tissues asymptomatically without causing outward signs of disease or overt negative symptoms (Arnold et al., 2007; Christian et al., 2016). Endophytic fungi are often considered to be beneficial to their host plants, because they may provide the plant with protection against pathogens (Arnold et al., 2003), enhance resistance of the host plant to insect herbivory (Tanaka et al., 2005), confer salt and heat tolerance to native grass species from coastal and geothermal habitats (Rodriguez et al., 2008), and promote plant root formation and shoot growth (Sun et al., 2010). However, Faeth and Sullivan (2003) challenged the notion that systemic endophytic fungi should form a mutually beneficial relationship with the host plant for infections to persist in nature. Some endophytic fungi are capable of pathogenicity under stressful conditions or have long latent periods (Arnold, 2007). Thus, the interaction between endophytic fungi and host plants can span the spectrum from mutualism to antagonism based on the genetic and/or environmental context (Vincent et al., 2016).

Rhizosphere, which is a narrow zone surrounding the plant roots, is a hot-spot of microbial activity and interactions (Raaijmakers, 2015; Wang et al., 2017). The rhizosphere microbial community harbors members that exert neutral, deleterious, or beneficial effects on their host plants, but are all part of the complex food web sustained by large amount of carbon and other nutrients released by plant roots (Raaijmakers et al., 2009; Raaijmakers, 2015). Therefore, the rhizosphere is akin to a battlefield where the complex microbial community members interact with each other and influence the outcome of pathogen infection and host health and growth (Raaijmakers et al., 2009). However, there is still limited understanding of the community structure and function of the rhizosphere fungal microbiome. Recently, based on high-throughput sequencing data, Zimudzi et al. (2018) suggested that the dominant rhizosphere fungal taxa potentially play either positive or negative functional roles, but that none of the dominant taxa are “ecological passengers.” Hence, similar to fungal endophytes, rhizosphere fungi are also essential for plant fitness and indirectly affect the composition and functioning of natural plant communities (Rudgers et al., 2012; Jogaiah et al., 2013; Philippot et al., 2013). Understanding the composition, dynamics, and activity of the endophytic and rhizosphere fungal community is therefore critical for the development of new strategies to promote plant growth and health in both agro-ecosystems and natural ecosystems (Raaijmakers et al., 2009).

Network analysis of taxa co-occurrence patterns revealing potential biotic interactions, habitat affinities, or shared physiologies among members, can offer new insights into the structure and assembly of complex microbial communities that cannot be obtained by the traditional suite of analytical approaches (Barberan et al., 2012; Zhang et al., 2018). Recently, with the advent of next generation sequencing, network analysis has been applied to explore microbial interaction patterns across a wide variety of habitats including soil (Ma et al., 2016; Rebollar et al., 2017; Li and Wu, 2018), water (Milici et al., 2016; Hu et al., 2017; Mikhailov et al., 2018), activated sludge (Ju et al., 2014), and even the human gut (Baldassano and Bassett, 2016; Jackson et al., 2018). In plant–microbe research, niche differentiation of microbial co-occurrence patterns between rhizosphere and bulk soil has attracted much attention (Mendes et al., 2014; Shi et al., 2016; Fan et al., 2018). However, compared with the plant-soil interface, we still lack basic understanding of co-occurrence patterns of fungal communities present in different plant compartments especially the leaf and root endospheres.

Here, we explore the fungal community composition and co-occurrence patterns associated with the rhizosphere, root and leaf endospheres of *Mussaenda kwangtungensis* H. L. Li (Rubiaceae) using next-generation sequencing of the fungal internal transcribed spacer 2 (ITS2) region. We hypothesized that due to distinct microenvironment filtering and different microbial inoculum sources, fungal community composition and network structure would differ significantly among the rhizosphere soil, root endosphere, and leaf endosphere compartments. *M. kwangtungensis* is a drought-resistant shrub that has historically been used in Traditional Chinese Medicine as an antichloristic and antipyretic agent against laryngopharyngitis, acute gastroenteritis, and dysentery. Endophytic and rhizosphere fungi are rich and relatively unsampled sources of novel bioactive compounds that could be used to produce new, promising drugs. Therefore, this study will not only advance our understanding in assembly principles and ecological interactions of plant associated fungal communities, but also may lay the foundation for further exploitation and optimization of the community for human advantage.

## Materials and Methods

### Study Site and Sampling

This study was conducted on the Dajia Island (22.57 N, 114.65 E), Guangdong Province, China. Dajia Island has a subtropical continental climate, with a mean annual temperature of 22.3°C and a mean annual precipitation of 1925 mm. *M. kwangtungensis* H. L. Li is distributed naturally and widely in the study area, which has no obvious environmental gradients. In September 2017, we selected ten healthy individual shrubs (>20 m apart from each other) that were in anthesis.

To investigate foliar fungal endophyte communities, we collected a random sample of nine mature and asymptomatic leaves from the middle of three current-year shoots from each plant. Leaves from the same shrub were placed in individual sterilized polyethylene bags and stored in coolers equipped with ice packs. All leaves were surface sterilized within 48 h after sampling to remove the presence of surface microorganisms. The surface sterilization was processed by consecutive immersion for 30 s in sterile water, 1 min in 75% (vol/vol) ethanol, 3 min in 3.25% sodium hypochlorite, and 30 s in 75% (vol/vol) ethanol. Sterilization was completed with three sequential 2 min rinses in sterile water. The leaves belonging to the same individual shrub were dried with sterile absorbent paper and pooled before grinding with liquid nitrogen. To validate the effectiveness of surface sterilization, the leaf surfaces were placed in each 90 mm Petri dish containing malt extract agar (MEA, 2%) and cultured in the dark for 48 h at 25°C to check for the appearance of colonies.

To investigate rhizosphere soil and root fungal communities, we extracted primary root samples at a depth of 5–10 cm below ground, following Beckers et al. (2016, 2017). Sterilized gloves, scissors, spades, and brushes were used to collect the samples, and these tools were washed with distilled water and wiped with 70% (vol/vol) ethanol at each sampling time to avoid cross contamination. A minimum of 10 g of roots was collected from three directions per individual plant. The soil that remained tightly adhered to the root surface was defined as rhizosphere soil (Fan et al., 2018). Therefore, after shaking off the loosely bound soil, root samples were placed into 50 mL sterile centrifuge tubes and were washed with 10 mM PBS buffer (130 mM NaCl, 7 mM Na_2_HPO_4_, 3 mM NaH_2_PO_4_, pH 7.4) on a shaking table (80 rpm) for 15 min. The soil particles directly dislodged from the roots represented the rhizosphere samples. Rhizosphere soil samples were then pelleted by centrifugation (5000 ×*g* for 15 min) in 50 ml tubes. The root samples were then separated, surface-sterilized and verified as described above for leaf samples. We finally pooled the separate samples from leaves, roots, and rhizosphere soil from the same individual because our primary interest was in variation of different communities across different compartments instead of variation between individuals. In total, we collected 30 samples (10 individual plants × 3 compartments) for DNA Extraction, and all the samples were stored at -80°C until processing.

### DNA Extraction, PCR Amplification, and Illumina Sequencing

We homogenized the leaf and root samples mixed with liquid nitrogen using sterilized mortars and pestles under aseptic conditions in a laminar airflow to avoid external contamination. We extracted total DNA from 500 mg of each sample (rhizosphere soil, root, and leaf) using the PowerSoil DNA Isolation kit (Mo Bio Laboratories, Carlsbad, CA, United States) following the manufacturer’s instructions. DNA quality and quantity were assessed on a NanoDrop 1000 spectrophotometer (Thermo Scientific, Wilmington, DE, United States).

We used a two-step PCR approach to prepare amplicon libraries for the Illumina HiSeq sequencing platform. First, we amplified the entire fungal ITS region using conventional primers ITS1F (Gardes and Bruns, 1993) and ITS4 (White et al., 1990). The first round of PCR amplification was performed in 25 μL reaction volumes including 250 μM of dNTP Mix, 1 unit Phusion DNA polymerase (New England Biolabs, Hitchin, United Kingdom), 0.5 μM of each primer, 2.5 μL Phusion HF Buffer and c. 10 ng DNA template. Cycling conditions included 94°C for 3 min, followed by 25 cycles of 94°C for 50 s, 53°C for 50 s, and 72°C for 1 min, and a final extension of 72°C for 10 min. Second, we performed a second round of PCR amplification with primer fITS7 (Ihrmark et al., 2012) and reverse primer ITS4 to target the fungal ITS2 region. Both of the primers were amended with Illumina Nextera transposase sequence. PCR conditions were identical to those described above, except for the cycle number, which was lowered to 20. To minimize PCR biases, each sample was amplified in triplicate and the replicate products were pooled together into a general sample. We included negative controls (replaced DNA template by ddH_2_O) to assess the presence of contaminating sequences during the DNA extraction and PCR process, which were checked by gel electrophoresis. PCR products were then cleaned and purified using a MinElute PCR Purification Kit (Qiagen, Venlo, Netherlands). Each cleaned PCR product from the same sample received a unique combination of forward and reverse primers from the Illumina Index Kit PCR primers to add a dual index system. The PCR reaction to add index and sequencing adapters was performed in 25 μL reaction volumes including 3 μL of Illumina N7xx oligo, 3 μL of Illumina S5xx oligo and 13 μL of HiFi ReadyMix. Thermal cycling conditions were as follows: 94°C for 5 min, followed by 12 cycles of 98°C for 20 s, annealing at 55°C for 30 s and 68°C for 30 s. The resulting products were then purified using Agencourt purification beads (Beckman Coulter, Brea, CA, United States). We quantified the sequencing libraries employing the Kapa qPCR-based quantification kit (Kapa Biosystems, Boston, MA, United States). The library concentration was adjusted to 14 pM. We clustered the libraries according to Illumina protocol and sequenced the libraries by an Illumina HiSeq 2500 platform using the paired end option (2 bp × 250 bp) at Biomarker Technologies Co., Ltd., (Beijing, China). The Illumina sequences were deposited in the NCBI Sequence Read Archive (accession number SRP156485).

### Bioinformatics Workflow

We processed the raw data with Trimmomatic (Bolger et al., 2014) to trim and filter adapters, primer sites, and low quality ends of reads. The overlapping paired-end reads were merged to a single sequence using FLASH (Magoc and Salzberg, 2011). Chimera sequences were detected and removed using the UCHIME program (Edgar et al., 2011) referencing the UNITE database^1^. We then employed the Uclust algorithm (Edgar, 2010) to bin the non-chimera sequences that passed the filtering processes into the operational taxonomic units (OTUs) at a 97% sequence similarity cutoff. Further, the taxonomic classification of each OTU was carried out using RDP Classifier (Wang et al., 2007) with a confidence threshold of 0.8. The taxonomic identity of each OTU was determined based on a BLAST search against the UNITE reference database (see text footnote 1). We excluded the OTUs with less than 10 reads in all samples as their sequences may contain PCR or sequencing errors (Eusemann et al., 2016). To eliminate the effects of sequence number variation from different samples, we rarefied each sample to the minimum sequencing depth through a subset of randomly selected reads prior to downstream analysis. This rarefaction was performed by the “sub.sample” command in MOTHUR (Schloss et al., 2009).

### Statistical Analysis

We used QIIME scripts (Caporaso et al., 2010) to plot and calculate a rarefaction curve, rank abundance curve, and alpha diversity index including ACE value (Chao and Lee, 1992), Chao1 (Chao, 1984), Shannon index (Shannon, 2001), Pielou’s evenness (Pielou, 1966), and Good’s coverage (Good, 1953) for each compartment. We then performed one-way analysis of variance (ANOVA) and subsequent *post hoc* Tukey’s honestly significant difference (HSD) tests to compare differences in alpha diversity across different plant compartments. To investigate the patterns of fungal community structure, we performed nonmetric multidimensional scaling (NMDS) ordination and principle coordinate analysis (PCoA) with the Bray–Curtis and Jaccard distance calculated from the OTU community matrix, respectively. The Bray–Curtis matrix was also used to perform hierarchical clustering analysis of different compartments. Further, we conducted the mean nearest taxon distance (MNTD) dividing the fungal communities into two groups to represent the phylogenetic beta diversity indices (Stegen et al., 2012; Yang et al., 2016). We tested the difference in the fungal community composition among various plant habitats by conducting three different permutation tests including permutational multivariate analysis of variance (ADONIS), analysis of similarity (ANOSIM), and multiple response permutation procedure (MRPP). To identify OTUs that are correlated with community separation between different compartments, we performed differential OTU abundance analysis using a generalized linear modeling (GLM) approach following Edwards et al. (2015). The statistical analyses mentioned above were performed with vegan (Oksanen et al., 2016), ape (Paradis et al., 2004), picante (Kembel et al., 2010), and edgeR (Robinson et al., 2010) packages in R 3.4.3^2^ (Ihaka and Gentleman, 1996).

We used the FUNGuild data base (Nguyen et al., 2016) to assign each OTU to an ecological guild to examine if fungal functional groups differed among plant compartments. We only retained OTUs with a confidence ranking of “highly probable” or “probable” in our analysis as per Cregger et al. (2018). Venn diagrams of each class of trophic mode were visualized using the VennDiagram R package (Chen and Boutros, 2011). We also plotted ternary diagrams using ternary python scripts and a network-like Venn diagram using the Cytoscape program to show the distribution of total fungal OTUs across different habitats (Shannon et al., 2003). Furthermore, we employed Linear Discriminant Analysis (LDA) effect size (LEfSe^3^; Segata et al., 2011) to test the significant taxonomic difference among the three compartments. A logarithmic LDA score of 4.0 was set as the threshold for discriminative features.

To better understand the connectedness within the fungal communities, we constructed the co-occurrence networks by calculating all possible Spearman’s correlation coefficients between OTUs across different compartments. We corrected *P*-values for multiple testing using the false discovery rate (FDR) according to Benjamini and Hochberg (1995). We considered a valid relationship to be statistically robust if the rank correlation coefficient *r* > |0.6| and if it was significant at *P* < 0.05 (Widder et al., 2014). The network of each compartment was visualized using the software Gephi (Bastian et al., 2009). The nodes in the networks represent fungal OTUs, and the edges correspond to strong and significant correlations among nodes (Mendes et al., 2014). Meanwhile, 10000 Erdös–Rényi model random networks (Erdös and Rényi, 1960), which had the same number of nodes and edges as the observed networks, were constructed for each compartment. We employed the igraph package (Csardi and Nepusz, 2006) to test whether the network degrees fit a power-law distribution (Clauset et al., 2009) with the Kolmogorov–Smirnov test. We then calculated a set of network parameters including average path length, clustering coefficient, number of clusters, and modularity for both observed networks and random networks to investigate their structure complexity and topology characteristics. Node-level topological properties (betweenness and degree) were also calculated in the same package to compare the differences in measured node-level attributes across different compartments according to Xue et al. (2018) and to determine the keystone OTUs in each network with high degree (>100) and low betweenness centrality values (<5000) following Ma et al. (2016) and Xue et al. (2018).

## Results

### Fungal Community Diversity

High-throughput Illumina sequencing resulted in a total of 2,424,236 raw reads. Quality filtering reduced the dataset to 2,301,895 high quality reads. These reads were clustered into 2490 OTUs at a 97% sequence similarity level. We removed OTUs that have fewer than 10 reads, and kept 2,242,941 fungal ITS2 reads. As the remaining numbers of reads ranged from 45,787 to 114,146 among samples, we rarefied each sample to the minimum size (45,787), resulting in a normalized dataset comprising 1,542 fungal OTUs (from 1,373,610 high-quality sequences). We found an average of 388 ± 184 OTUs (mean ± sd) per sample, with 156 ± 32 OTUs per leaf endophyte sample, 430 ± 53 OTUs per root endophyte sample, and 579 ± 57 OTUs per rhizosphere soil sample.

**FIGURE 1:**
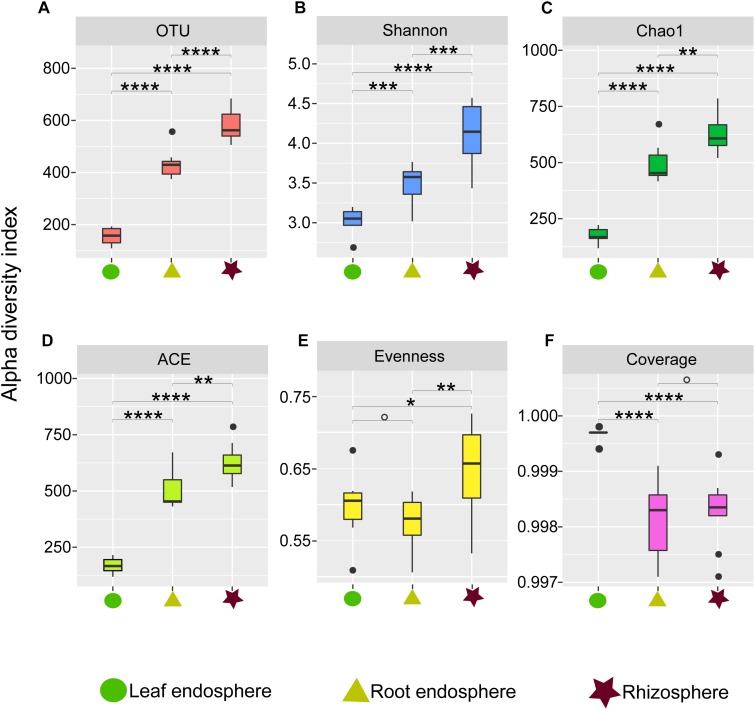
Alpha diversity estimates of the fungal communities in different plant compartments. **(A)** Number of observed OTUs. **(B)** Shannon diversity. **(C)** Chao1. **(D)** ACE value. **(E)** Pielou’s evenness. **(F)** Good’s coverage scores. One-way ANOVA with Tukey’s HSD tests were performed to reveal significant differences in alpha diversity among the plant compartments. ^O^, No significant; ^∗^*P* < 0.05; ^∗∗^*P* < 0.01; ^∗∗∗^*P* < 0.001; ^∗∗∗∗^*P* < 0.0001.

Our rarefaction curves of OTU richness and Shannon diversity per compartment reached a saturation plateau, suggesting that we had sampled the majority of the diversity in the *M. kwangtungensis* mycobiome. The shape of the curves demonstrated that the OTU richness and Shannon diversity was consistently highest in the rhizosphere community followed by that of the root endosphere (Supplementary Figure S1). Good’s coverage scores of all of the compartments were very high ranging from 99.82% ± 0.0218% to 99.97% ± 0.0004% (Figure 1F), which further indicated that the sequencing depths were sufficient to reliably describe the fungal microbiome. The slope of the rank abundance curve for the leaf endosphere was greater than the two belowground compartments, indicating a lower relative abundance within the leaf endophytic fungal community (Supplementary Figure S2).

Our measures of the alpha diversity within each compartment showed that OTU richness, ACE, Chao1, and Shannon diversity were all highly dependent on the compartment (Tukey’s HSD test: *P* < 0.01) with the highest fungal alpha diversity in the rhizosphere soil and consistently lower fungal diversity in the root endosphere and leaf endosphere compartments (Figure 1A–D). Similarly, we found higher Pielou evenness value in the rhizosphere soil samples compared to the leaf and root endosphere compartments (Figure 1E).

### Fungal Community Composition

**FIGURE 2:**
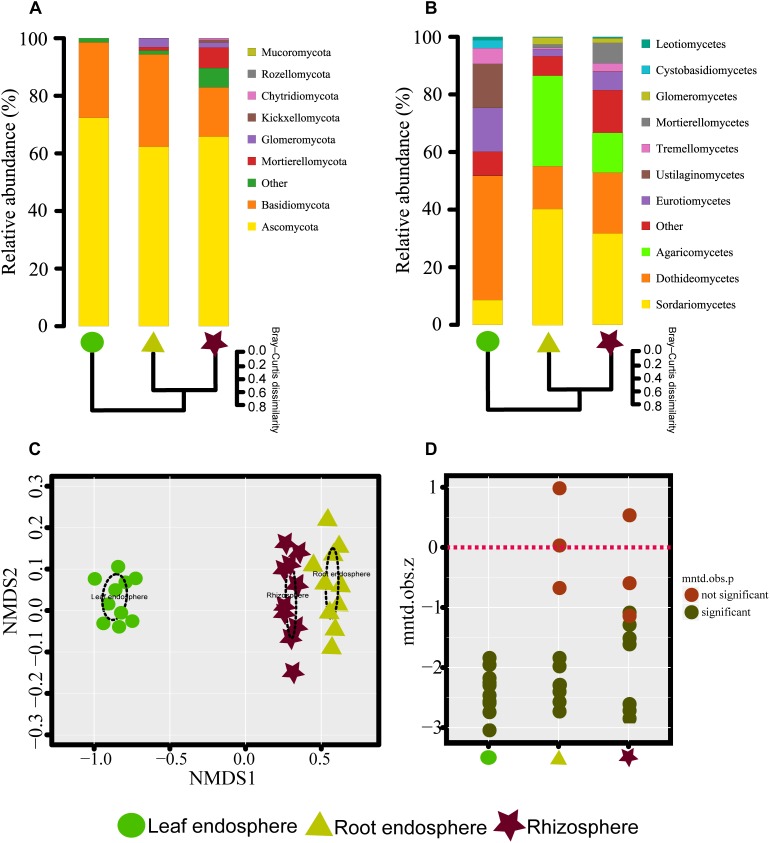
**(A,B)** Taxonomic composition of the fungal communities associated with *Mussaenda kwangtungensis* at the phylum and class level, respectively. The hierarchical clustering dendrograms based on Bray–Curtis dissimilarity are shown. **(C)** Nonmetric multidimensional scaling (NMDS) ordinations based on Bray–Curtis dissimilarity of fungal communities across the three habitat classifications. **(D)** Variation of standardized effect sizes of the mean nearest taxon distance among different compartments.

We were able to assign phylum to 87.03% of the OTUs. As expected, *M. kwangtungensis* associated fungal communities were dominated by the phyla Ascomycota (66.81% of all sequences) and Basidiomycota (24.37%), Neither phylum differed significantly in relative abundance across the three compartments (Wilcoxon test, *P* > 0.05). Other minor phyla such as Chytridiomycota, Glomeromycota, Kickxellomycota, Mortierellomycota, Mucoromycota, and Rozellomycota were mostly found in the belowground compartments (Figure 2A and Supplementary Figure S3A). Variation in community composition among the three plant compartments was also observed at the class level (Figure 2B and Supplementary Figure S3B). The three most abundant classes (Sordariomycetes, 26.9%; Dothideomycetes, 26.4%; and Agaricomycetes, 15.1%) showed different patterns of relative abundance. Dothideomycetes (Ascomycota) was most abundant in the leaf endosphere compared to belowground compartments (Wilcoxon test, *P* < 0.05), but did not differ significantly between the roots and rhizosphere. Conversely, Sordariomycetes (Ascomycota) had lower abundance in the leaf endosphere than in the root endosphere and rhizosphere (Wilcoxon test, *P* < 0.01), as were Agaricomycetes (Basidiomycota). In total, 288 genera were identified in our study. The top 10 genera for the three different plant compartments are listed in Supplementary Table S1. *Aureobasidium*, *Delicatula*, and *Mortierella* were the most abundant genera in leaves, roots and rhizosphere soil, respectively.

**FIGURE 3:**
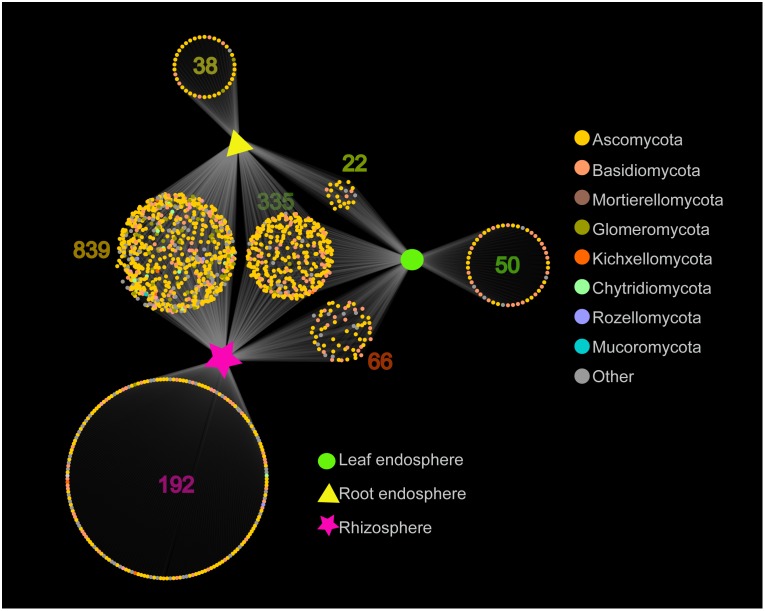
Network-like Venn diagram showing overlap and partitioning of the OTUs among the different plant compartments.

**FIGURE 4:**
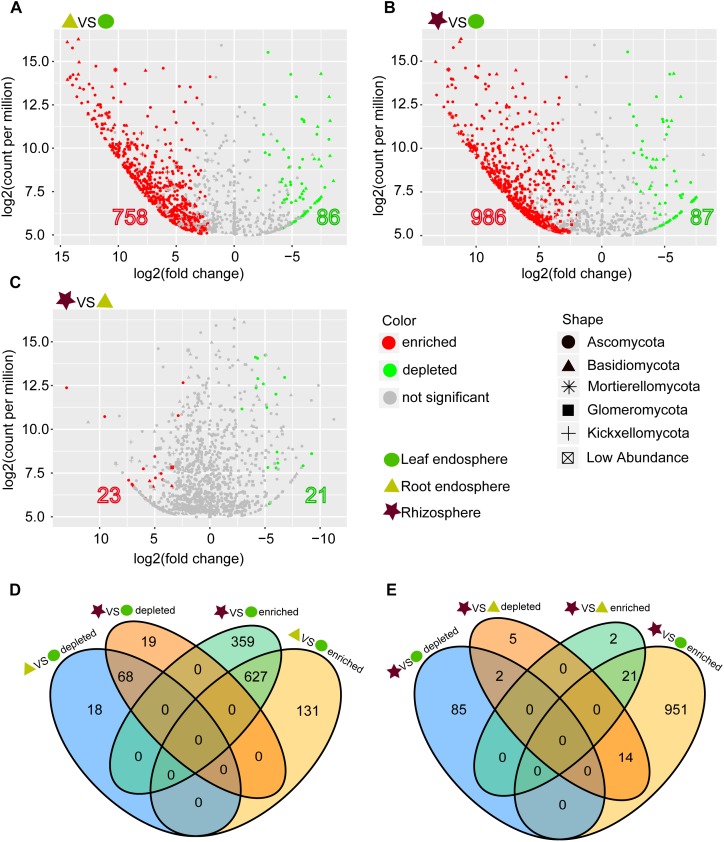
**(A–C)** Enrichment and depletion of the fungal OTUs in the three compartments compared with each other as determined by differential abundance analysis. **(D–E)** Numbers of differentially enriched OTUs and depleted OTUs between each compartment compared with each other.

The network-like Venn diagram showed the number of specific and shared fungal OTUs of *M. kwangtungensis* across different compartments (Figure 3). Shared OTUs (839) between the root endosphere and rhizosphere accounted for the largest component (54.4%) of total OTUs, followed by 335 OTUs (21.7%) shared across the three compartments. Pairwise OTUs coexisting in leaf and root endosphere accounted for the lowest proportion (1.4%). We also observed a relatively low number of compartment-specific fungal OTUs in all compartments: rhizosphere (12.5%), leaf endosphere (3.2%), and root endosphere (2.5%). Differential OTU abundance analysis using an adjusted *P*-value cutoff of 0.01 was then conducted to further classify OTUs that are significantly correlated with community separation among the three plant habitats (Figure 4). There were 758 and 986 fungal OTUs that were significantly enriched in the root endosphere and the rhizosphere, respectively, when using leaf endosphere as a control, (Figure 4A,B). The fungal communities in the two belowground compartments were much more similar with each other, as indicated by the lowest number of deleted and enriched OTUs and the relatively long insignificant tail (Figure 4C). It is worth noting that 627 out of the 758 fungal OTUs enriched in the root endosphere were also enriched in rhizosphere soil, when using the leaf endosphere as a control (Figure 4D). In contrast, only a small number of OTUs (21) were shared between enriched in rhizosphere vs. leaf endosphere and vs. root endosphere (Figure 4E).

**FIGURE 5:**
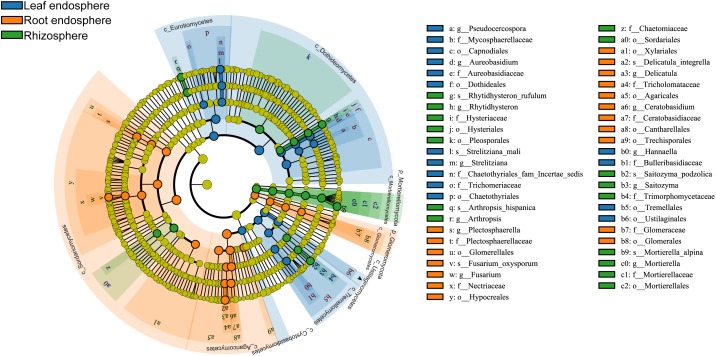
Cladograms of LEfSe showing fungal indicator taxa at each compartment. The filled circles from inside to outside indicate the taxonomic levels with phylum, class, order, family, genus, and species. Circles or nodes shown in color corresponding to different plant compartments represent a significant association.

Many fungal taxa at different taxonomic levels were significantly associated with different compartments (LEfSe analysis, Figure 5 and Supplementary Figure S5). The phyla Mortierellomycota and Glomeromycota appeared as the main discriminant phyla for the rhizosphere and root endosphere, respectively. Dothideomycetes, Eurotiomycetes, and Tremellomycetes were the three most abundant fungal classes in the leaf endosphere, while Sordariomycetes, Agaricomycetes, and Glomeromycetes were differentially enriched in the root endosphere. We detected a total of four indicator species [*Rhytidhysteron rufulum* (Spreng.) Speg., *Saitozyma podzolica* (Babeva & Reshetova) Xin Zhan Liu, F.Y. Bai, M. Groenew. & Boekhout, *Mortierella alpina* Peyronel, and *Arthropsis hispanica* Gené, Ulfig & Guarro] that were significantly associated with rhizosphere soil; two indicator species [*Fusarium oxysporum* sensu Smith & Swingle, and *Delicatula integrella* (Pers.) Fayod] associated with the root endosphere, and only *Strelitziana mali* Rong Zhang & G.Y. Sun was a significant indicator for the leaf endosphere.

The NMDS plots depicting fungal communities combined with hierarchical clustering analysis based on the Bray–Curtis dissimilarity revealed that there was a clear distinction among the three plant compartments. In addition, the plots indicated that rhizosphere soil communities clustered close to the root endosphere communities, while leaf endophytic samples were grouped separately (Figure 2A–C). In addition, the two belowground compartments exhibited relatively higher between-sample variation, while leaf endosphere samples clustered more closely together (Figure 2C,D). Similar patterns of clustering were also observed in the PCoA analysis using Jaccard distance (Supplementary Figure S4). The result of standardized effect size of MNTD (ses.MNTD) showed that the root endosphere and rhizosphere had a more similar phylogenetic community structure than the leaf endosphere did (Figure 2D). All of the ses.MNTD values from the leaf endosphere samples were significantly negative, indicating that the fungal communities in this compartment were more phylogenetically clustered than expected by chance. Three non-parametric multivariate statistical tests, which were performed with ADONIS, ANOSIM, and MRPP based on both Bray–Curtis dissimilarity and MNTD distance, further confirmed significant differentiation of fungal community structure between different compartments (Table 1).

**Table 1:** Fungal community dissimilarity comparison among different plant compartments using three non-parametric statistical methods.

		ADONIS		ANOSIM		MRPP	
	Multiple comparison	*R*^2^	*P*	*R*	*P*	δ	*P*
Bray–Curtis dissimilarity	Rhizosphere vs. Root vs. Leaf	0.399	0.001	0.8756	0.001	0.2035	0.001
	Rhizosphere vs. Root	0.141	0.001	0.4833	0.001	0.0480	0.001
	Rhizosphere vs. Leaf	0.411	0.001	0.9993	0.001	0.2144	0.001
	Root vs. Leaf	0.425	0.001	0.9978	0.001	0.2241	0.001
MNTD dissimilarity	Rhizosphere vs. Root vs. Leaf	0.902	0.001	0.8027	0.001	0.6707	0.001
	Rhizosphere vs. Root	0.301	0.008	0.2953	0.002	0.1547	0.004
	Rhizosphere vs. Leaf	0.932	0.001	0.9947	0.001	0.7235	0.001
	Root vs. Leaf	0.913	0.001	0.9722	0.001	0.6995	0.001


The seven main fungal trophic guilds (symbiotroph, pathotroph, saprotroph, symbiotroph-pathotroph, symbiotroph-saprotroph, saprotroph-pathotroph, and symbiotroph-saprotroph-pathotroph) were all detected in the analyzed plant compartments, and their abundance distribution showed greater or lesser extent of compartment-specificity (Supplementary Figure S6A). Pathotrophic fungi were the most abundant functional group (13.31% of all sequences), followed by saprotroph (6.49%), symbiotroph-pathotroph (5.46%), and symbiotroph (4.23%) groups. The OTU richness of trophic guilds was also different between habitats (Supplementary Figures S6B–H). The majority of saprotroph species existed in the compartment shared between the root endosphere and rhizosphere soil, as were the symbiotroph species, due to the majority of this guild being mycorrhizal fungi. We also observed many shared OTUs with unique or multiple functional guilds co-existing in all the three habitats.

### Fungal Networks

**FIGURE 6:**
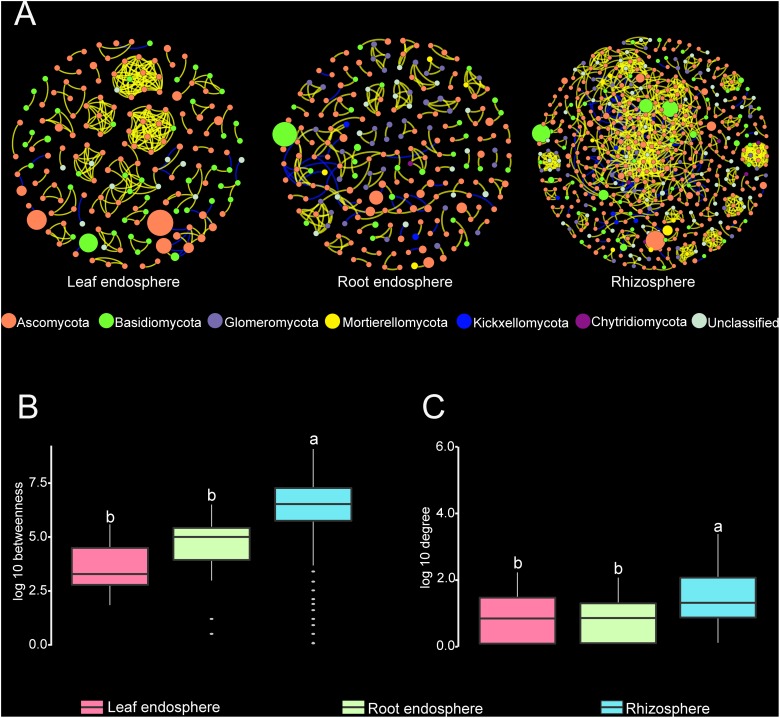
**(A)** Correlation-based networks of fungal communities in the leaf endosphere, root endosphere, and rhizosphere. Fungal OTUs are represented as nodes and significant correlations as edges. The node color indicates the corresponding taxonomic assignment at the phylum level. The size of each node is proportional to the OTU abundances and the color of each line reflects positive (yellow) or negative (blue) associations. **(B,C)** Comparison of node-level betweenness centrality and degree among the different compartments. Different letters indicate the significance level at *P* < 0.05.

Construction of correlation-based networks of the fungal communities resulted in three networks, consisting of 173, 219, and 517 nodes connected by 285, 230, and 1085 edges, respectively (Table 2 and Figure 6). The composition of nodes and edges differed strikingly within each network: no links and only ten nodes were shared in all three networks. Each network had a much higher number of strongly positive correlations (93.68, 87.39, and 86.36% in leaf endosphere, root endosphere, and rhizosphere network, respectively) than negative correlations. We found that we could not reject the null hypothesis that the network data were drawn from the fitted power-law distribution (Kolmogorov–Smirnov test, *P* > 0.05).

**Table 2:** Key topological properties of the fungal communities in the leaf endosphere, root endosphere, and rhizosphere.

Network properties	Leaf endosphere	Root endosphere	Rhizosphere
**Observed networks**			
Number of nodes	173	219	517
Number of edges	285	230	1085
Clustering coefficient	0.9302	0.7007	0.5564
Number of clusters	46	64	81
Average path length	5.6818	6.9502	7.3709
Modularity	0.8942	0.9293	0.8143
**Random networks**			
Clustering coefficient	0.0188 ± 0.0075	0.0096 ± 0.0078	0.0081 ± 0.0024
Number of clusters	7.5 ± 2.2570	32.12 ± 3.9784	8.772 ± 2.5998
Average path length	4.3181 ± 0.0884	6.4652 ± 0.3677	4.4797 ± 0.0279
Modularity	0.5468 ± 0.0124	0.7221 ± 0.0160	0.4933 ± 0.0056


The average path length, number of clusters, and clustering coefficient of the observed networks were all significantly higher than those of Erdös–Rényi random networks (Table 2). The values of network modularity were also higher than their corresponding random networks and larger than 0.4. Further, we found more modular structure in leaf and root endosphere networks due to their larger modularity values (Table 2). The node betweenness and degree were significantly larger for rhizosphere network than for the leaf and root endosphere networks (Figure 6B,C), suggesting lower centrality and connectedness in the two endosphere networks. Interestingly, no fungal OTU was a keystone taxon for any co-occurrence networks.

## Discussion

### Distinct Community Structures Among Compartments

We found that the leaf endosphere, root endosphere, and rhizosphere fungal communities differed significantly with all of the various indices of alpha diversity used in this study. Similar results were found for the bacterial microbiome of poplar trees (Beckers et al., 2017), which also showed that the rhizosphere harbored strikingly higher OTU richness than the endophytic compartments. The rhizosphere forms a highly active transition zone between the root surface and bulk soil, through root exudates, mucilage produced by the root caps, and the release of sloughed-off root cells, which all provide suitable ecological niches for the growth, development, and reproduction of microbial communities (Buée et al., 2009). In contrast, successful endophytic colonization involves the expression of genes associated with the production of cell-wall-degrading enzymes and resistance to a range of plant innate immune responses (Jones and Dangl, 2006; Hardoim et al., 2008). Furthermore, the microorganisms inhabiting leaf environments are constantly exposed to harsh conditions such as ultraviolet radiation exposure, low nutrient availability, and high temperature fluctuations throughout the diurnal rhythm (Remus-Emsermann and Schlechter, 2018). These environmental filters would suggest a lower diversity of leaf endophytic fungi than that in belowground habitats.

Additionally, the more phylogenetically clustered community structure we found in the leaf fungal communities further confirmed a filtering process allowing only specific fungal members to successfully colonize the inside of the leaf tissues. The markedly lower diversity and evenness between the rhizosphere to endophytic habitats suggest that relatively few microbial species can adapt to the endophytic environment and these microorganisms will therefore dominate the endophytic communities (Beckers et al., 2017).

We also found clear separations among the fungal community composition of the three plant compartments, indicating that different micro-environments may be a main driver for selection (Fonseca-García et al., 2016). Such differences between compartments in fungal communities were also found in species of *Agave* (Coleman-Derr et al., 2015), *Salix* (Tardif et al., 2016), Cactaceae (Fonseca-García et al., 2016), and *Betula* (Yang et al., 2016). The fungal microbiome of the leaf endosphere was mainly comprised of Ascomycota taxa and dominated by members of Dothideomycetes, which was also the indicator fungal class for leaf endophytes. These taxa were previously found within leaves via high-throughput sequencing. For instance, Zimmerman and Vitousek (2012) found that Ascomycetes were the most abundant endophytic fungi recovered from *Metrosideros polymorpha* Gaudich. leaves, with major fungal reads assigned to the Dothideomycetes. Kemler et al. (2013) also revealed a dominance of Ascomycota abundance and especially of Dothideomycetes taxa in the leaf endophytic fungal community of *Eucalyptus grandis* W.Hill. Similar observations have also been reported in the phyllosphere fungal communities of *Sequoia sempervirens* (D.Don) Endl. (Harrison et al., 2016), *Hopea ferrea* Laness. (Izuno et al., 2016)*, Picea glauca* (Moench) Voss (Eusemann et al., 2016) and some mangrove species (Yao et al., 2019). Interestingly, we also detected abundant Basidiomycota leaf endophytes, which were often absent from previous studies based on culturing-dependent methods. High-throughput sequencing thus provides us the capability to detect uncultivable taxa (Rajala et al., 2014; Eusemann et al., 2016). The most abundant genus (*Aureobasidium*) in *M. kwangtungensis* leaf has been known as a group of black yeasts with high ability of stress-tolerance, which can help host plant to withstand high ultraviolet (UV) radiation and moderately osmotic condition in the island environment (Li et al., 2015; Turk and Gostinčar, 2018). It is noteworthy that *S. mali*, which has been documented as a plant pathogen (Zhang et al., 2009), was significantly enriched in the leaf endosphere. Several studies have addressed the issue that some fungal endophytes may be latent pathogens (Carroll, 1988; Photita et al., 2004). Many plant pathogens such as *Zymoseptoria tritici* (Roberge ex Desm.) Quaedvl. & Crous, *Microdochium nivale* (Fr.) Samuels & I.C. Hallett, and *Ramularia collo-cygni* B. Sutton & J.M. Waller have also been classified as indicator fungal species inhabiting the cereal phyllosphere habitat (Sapkota et al., 2015). Many fungal OTUs were shared between the leaf endosphere and the two belowground compartments, which could be explained by the horizontal transmission of these endophytic fungi that may originate from the soil, which serves as a primary reservoir for potential plant-associated fungi, through the roots to aboveground parts (Zarraonaindia et al., 2015). This hypothesis was supported by the differential OTU abundance analysis, showing that the belowground compartments played an enriching role for the plant associated fungal taxa relative to the leaf endosphere. Additionally, some endophytic fungi may also colonize the leaf surface via aerosols and subsequently penetrate into the leaf endosphere as evidenced by fungal OTUs uniquely found in the leaf endosphere.

In addition to the rhizosphere fungal microbiome, various ecological guilds could also be detected in the leaf and root endosphere mycobiome, consistent with a recent study on the fungal endophyte community of *Fagus sylvatica* L. (Siddique et al., 2017). Thus, plant fungal endophytes might involve a much wider functional range than previously thought. For example, several saprotroph or saprotroph-containing groups in the plant endosphere, usually act as the primary colonizers, initiating decomposition of cellulose in senescent leaves and young litter; they also play an important role in nutrient cycling and in the functional coupling of terrestrial ecosystems (Vacher et al., 2016).

In the belowground habitats, Glomeromycota was identified as the indicator phylum in the root endosphere. These ancient fungi have coevolved with plants for the last 400 million years and are the typical root symbiont (arbuscular mycorrhiza) in ∼80% of land plants (Bonfante and Genre, 2008; Davison et al., 2015). Arbuscular mycorrhizal fungi (AMF) can provide mineral nutrients to their plant hosts in exchange for reduced carbon (Rich et al., 2017), and could also act as a biostimulant that help host plant to enhance salt tolerance in island habitat (Zhang et al., 2017). In addition, the most abundant genus found in rhizosphere soil of *M. kwangtungensis* is *Mortierella* spp., which could interact with AMF to alleviate the deleterious effects of salt on plants growth and soil enzyme activities (Zhang et al., 2011). The indicator phylum in the rhizosphere compartment was Mortierellomycota, which was formerly classified to Zygomycota (Geml, 2018) and is usually found in the soil (Anslan et al., 2018). The physical proximity and close connection between root endosphere and rhizosphere soil leads to a considerable overlap in fungal community members and more similar community structure and phylogenetic relatedness across those two belowground compartments. Roots can recruit microbial members from the rhizosphere zone through a selective barrier (the root–soil interface) (Hardoim et al., 2008; Compant et al., 2010). Differential OTU abundance analysis showed that the vast majority of OTUs enriching the root endosphere was simultaneously enriched in the rhizosphere soil relative to leaf endophytic communities. These findings support a recruitment model in which factors induced by the roots attract fungal microorganisms that can then successfully colonize into the endosphere compartments (Edwards et al., 2015). The shared OTUs with unique or multiple functional guilds co-existing in multiple habitats indicate their highly complex life history and strong dispersal capability (Yang et al., 2016).

### Fungal Networks

Analysis of co-occurrence patterns can offer an in-depth and unique insight into the interactions between plant associated fungal communities and corresponding ecological assembly rules beyond those of community diversity (Xue et al., 2018). We found that the interrelationships between fungal OTUs in each network were predominantly positive, which suggested the potential for extensive cooperative and syntrophic interactions between most fungal taxa in their respective micro-environment. Similarly, this has also been observed in microbial networks in the bulk soil and rhizosphere habitats of wild oats (Shi et al., 2016), in mosses, lichens and the bark of maple trees (Aschenbrenner et al., 2017), and in the ocean surface (Chow et al., 2014). However, Deng et al. (2016) found a greater proportion of negative correlations in groundwater bacterial networks, suggesting that emulsified vegetable oil injections triggered fierce and intense competition among different bacterial species.

We showed that fungal co-occurrence networks have considerably different structure properties in the rhizosphere, leaf endosphere, and root endosphere. The niche differentiation among these plant compartments with substantially distinct micro-environments can explain this topological distinction. In the rhizosphere, the nutrients including volatile organic compounds, trace elements, and other metabolites deposited by plant roots are able to attract abundant microorganisms including fungi, making the rhizosphere to be one of the most dynamic interfaces on the earth (Philippot et al., 2013; Raaijmakers, 2015). Resource and food availabilities are important drivers of both macro- and microbiological network structures (Zhou et al., 2011; Foster et al., 2012). Therefore, the observation that rhizosphere soil was the most complex and connected compartment should come as no surprise. In addition, Shi et al. (2016) also suggested that some of the covariations detected represent guilds or niche-sharing within the gradients surrounding the roots, and could also be interpreted as evidence for the strong centrality and high complexity of the rhizosphere compartment.

The habitat-specific networks constructed here for fungal communities exhibited a highly modular architecture. One possible explanation for this structure feature is the lack of keystone taxa (no keystone species were detected in any of the three networks), which usually play disproportionately important roles in maintaining network structure relative to the other taxa in the network structure. The disappearance of these keystone taxa may segregate networks into more modules (Shi et al., 2016). Modules within networks could be considered as functional units due to niche partitioning under some conditions (Eiler et al., 2011; Wu et al., 2016). The modularity values of leaf and root endosphere networks were higher than that in the rhizosphere, which may be attributed to the more heterogeneous and disconnected substrate architecture in leaf and root tissues compared to rhizosphere soil. In a typical dicotyledon plant species, the leaf endosphere tissues are largely composed of xylem, phloem, palisade cells, and spongy mesophyll cells, while the cortical layer and vascular tissues are the main components of the root endosphere. This greater partitioning into higher congested, compartmentalized and separate environments may not only reduce the diversity of fungal community but also decrease the interactions between endophytic fungal members, resulting in less complex and tangled and more modular and fragmented networks of the leaf and root endosphere habitats.

In summary, our study characterized the fungal microbiome associated with *M. kwangtungensis* in the rhizosphere, leaf, and root endospheres using community profiling of ITS2 amplicons. We demonstrated that the associated fungal assemblages possessed high diversity in taxonomic classification and trophic guilds, and that the fungal diversity, community composition, and topological properties of the co-occurrence network varied significantly across different compartments. In particular, the rhizosphere fungal microbiome showed higher taxonomic diversity, and a more complex and connected network, than the plant endophytic microbiome did. This study provides a holistic perspective of the co-occurrence patterns, structure, and assembly of fungal communities found on and within plants, which will contribute to a better understanding of how the structural processes, interaction and relationships within and between mycobiomes impact the fitness and function of the overall plant holobiome. However, amplicon analysis does not offer any direct information of the natural history or life cycles of detected fungi (Toju et al., 2016). Therefore, further investigation using other technologies, such as fluorescence *in situ* hybridization, confocal laser scanning microscopy, metagenome or metatranscriptome approaches will be necessary to deeply understand niche differentiation and interspecific interactions in plant associated fungal communities.

## Author Contributions

XQ conceived and performed the experiments, analyzed the data, and drafted the manuscript. HL, YW, BW, MW, LC, XL, YiZ, XW, and MS performed the experiments and processed Illumina sequencing data. YoZ, LG, and DZ designed the study and approved the final draft.

## Conflict of Interest Statement

The authors declare that the research was conducted in the absence of any commercial or financial relationships that could be construed as a potential conflict of interest.

## Footnotes

1 https://unite.ut.ee/

2 https://www.r-project.org/

3 http://huttenhower.sph.harvard.edu/galaxy/

## Supplementary Material

FIGURE S1 Rarefaction curves of OTU richness and **(B)** Shannon index of fungal microbiome at each plant compartment.

FIGURE S2 Rank abundance curves for each plant compartment.

FIGURE S3 Ternary plots showing the distribution pattern of the fungal communities at the **(A)** phylum level and **(B)** class level. Each point represents an OTU, with its size corresponding to the abundance of the OTU.

FIGURE S4 Principal Coordinate Analysis (PCoA) using Bray–Curtis dissimilarities of fungal communities associated with *Mussaenda kwangtungensis*. **(A)** PCoA plot of first axis and second axis and **(B)** PCoA plot of second axis and third axis.

FIGURE S5 Histogram of the LDA scores computed for differentially abundant fungal taxa for each plant compartment.

FIGURE S6 Trophic guilds of fungal communities in the three plant compartments. The trophic guilds were assigned through the FUNGuild data base. **(B–H)** Venn diagrams of the seven trophic guilds among compartments. Green, pink, and blue circles represent leaf endosphere, root endosphere and rhizosphere, respectively. Sy, symbiotroph; Pa, pathotroph; Sa, saprotroph; Sy-Pa, symbiotroph-pathotroph; Sy-Sa, symbiotroph-saprotroph; Sa-Pa, saprotroph- pathotroph; Sy-Sa-Pa, symbiotroph-saprotroph-pathotroph.

TABLE S1 The 10 most abundant genera for the three different plant compartments.
