# Supplementary figures and images for: Leaf and Root Endospheres Harbor Lower Fungal Diversity and Less Complex Fungal Co-occurrence Patterns Than Rhizosphere
Source: Front Microbiol. 2019 May 8;10:1015. doi: 10.3389/fmicb.2019.01015 (PMC6521803; doi:10.3389/fmicb.2019.01015)

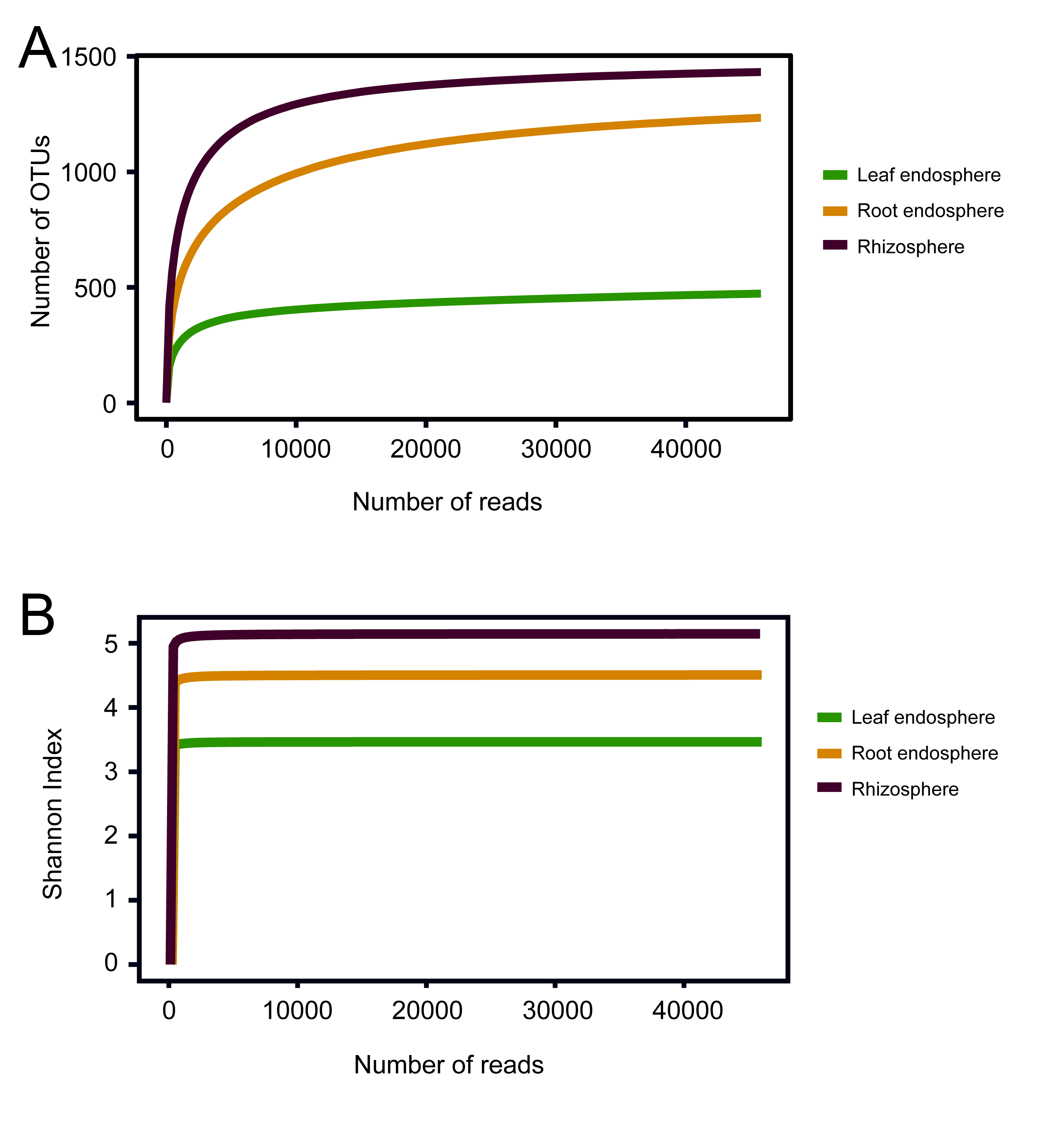

Supplement: FIGURE S1 — Rarefaction curves of OTU richness and (B) Shannon index of fungal microbiome at each plant compartment. [file Image_1.tif]

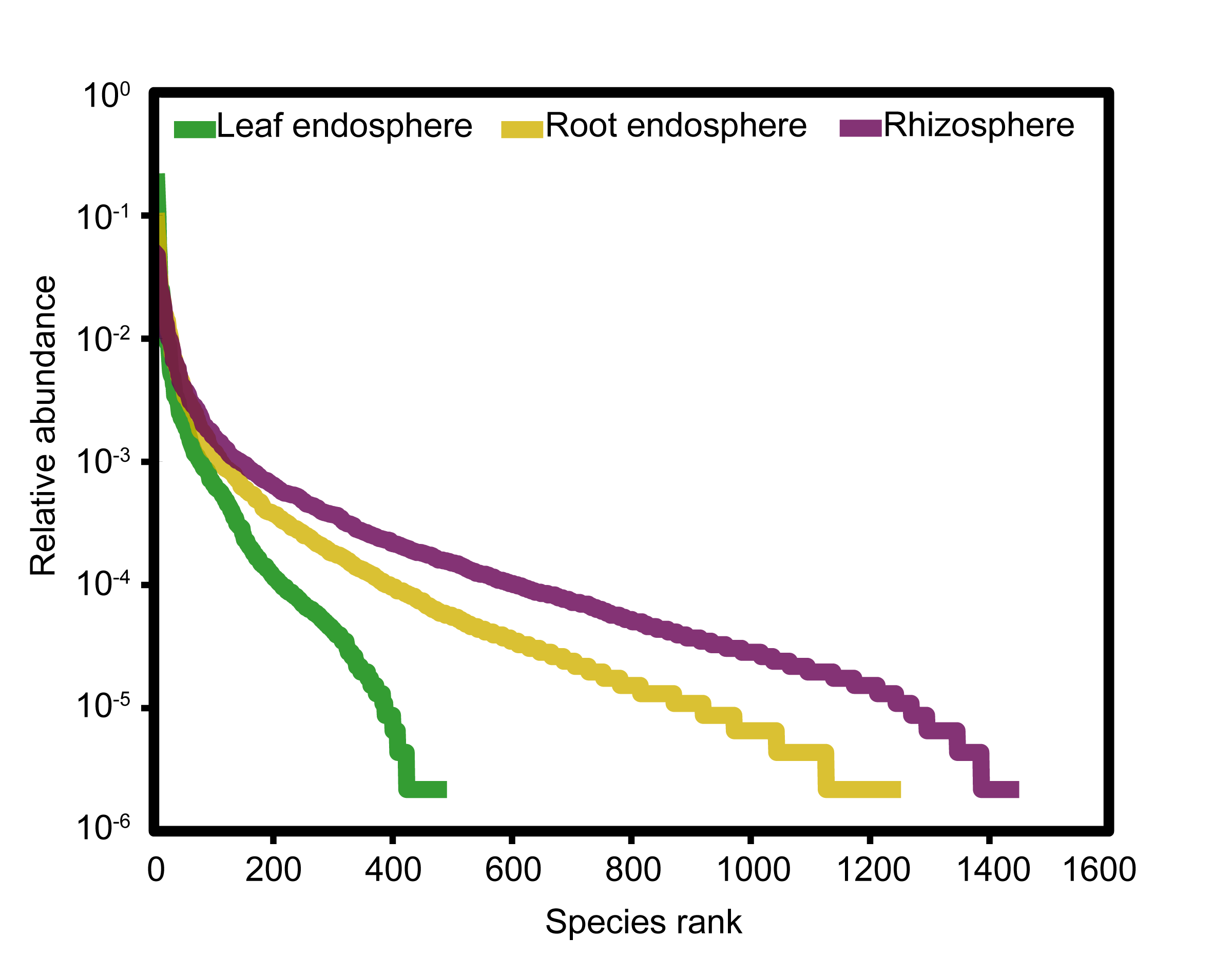

Supplement: FIGURE S2 — Rank abundance curves for each plant compartment. [file Image_2.TIF]

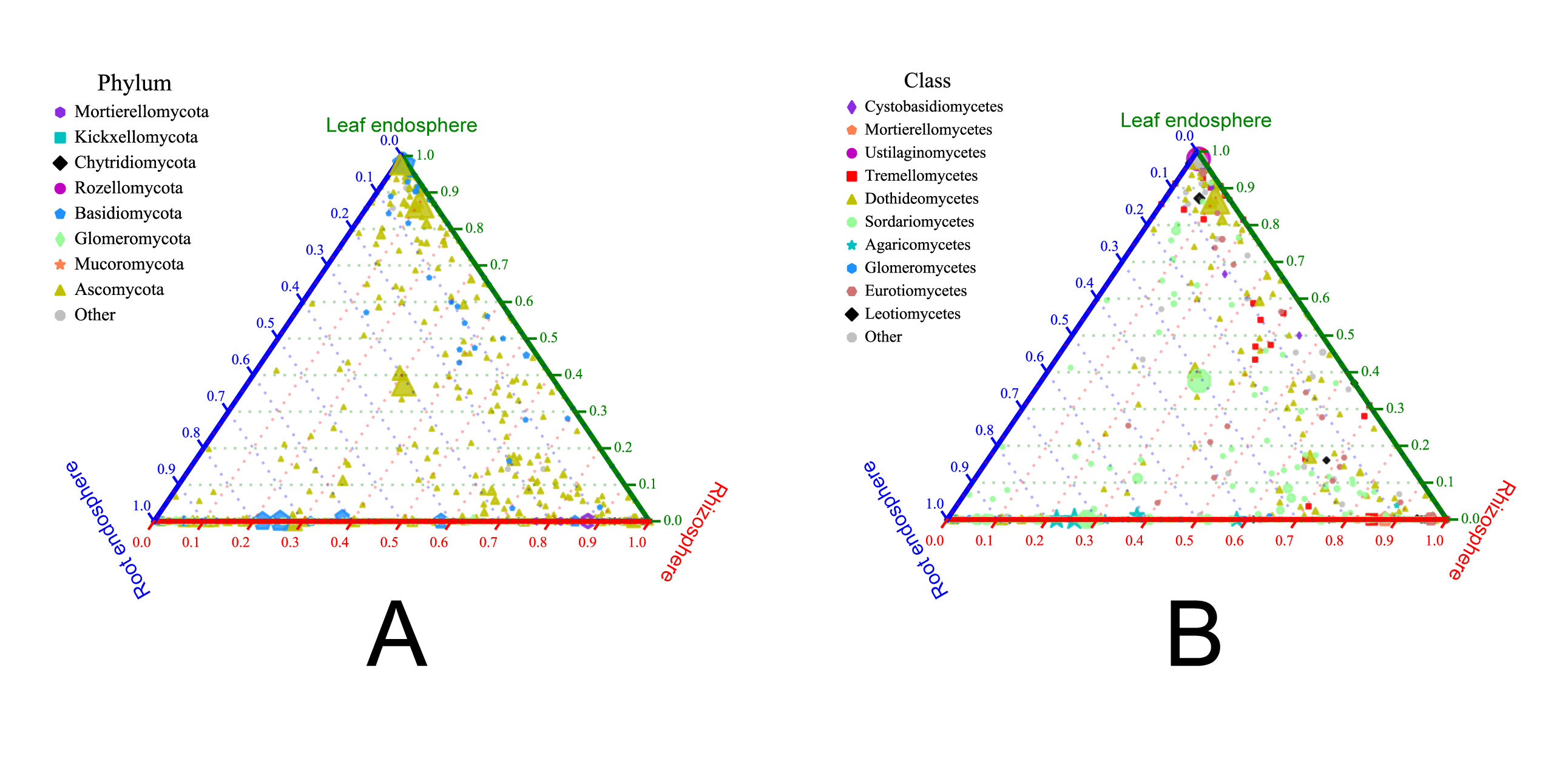

Supplement: FIGURE S3 — Ternary plots showing the distribution pattern of the fungal communities at the (A) phylum level and (B) class level. Each point represents an OTU, with its size corresponding to the abundance of the OTU. [file Image_3.TIF]

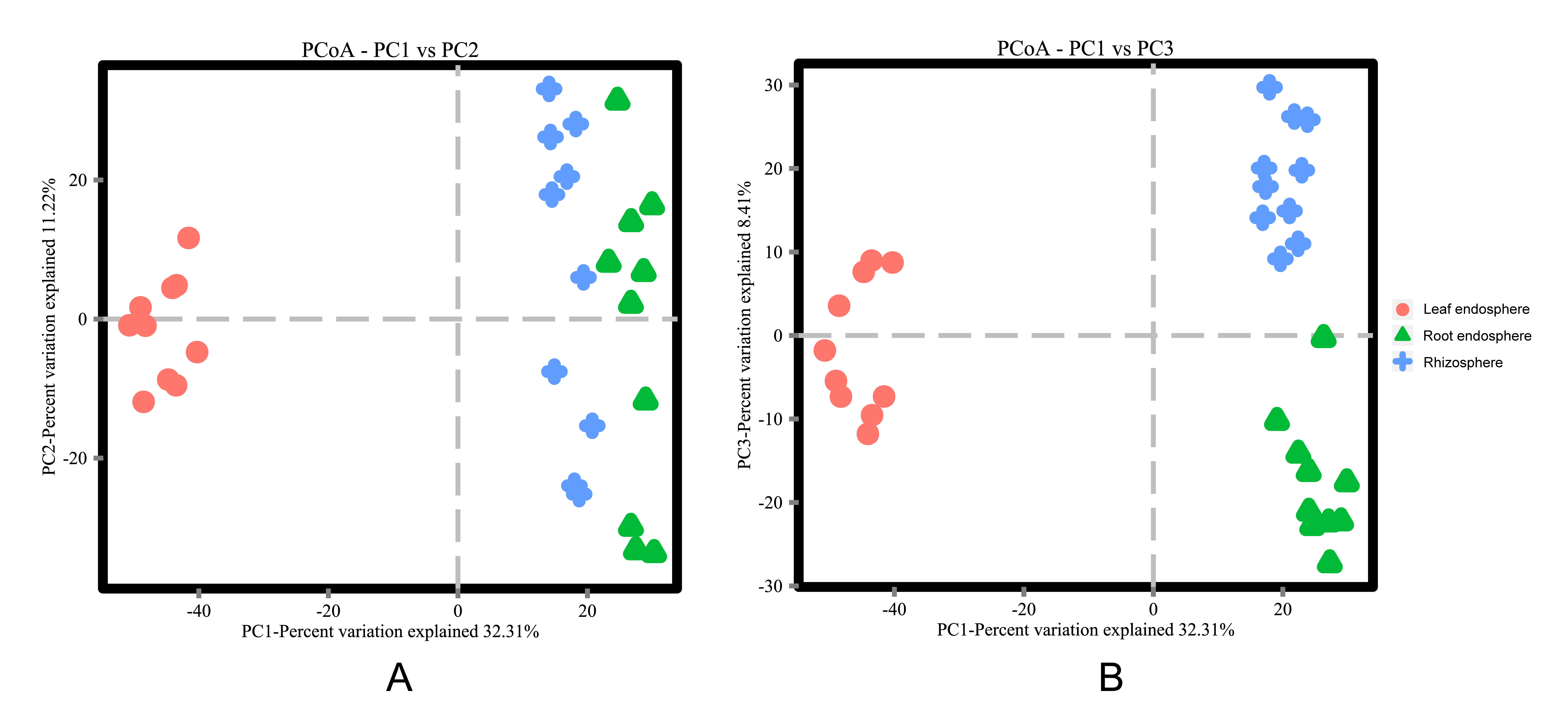

Supplement: FIGURE S4 — Principal Coordinate Analysis (PCoA) using Bray–Curtis dissimilarities of fungal communities associated with Mussaenda kwangtungensis. (A) PCoA plot of first axis and second axis and (B) PCoA plot of second axis and third axis. [file Image_4.TIF]

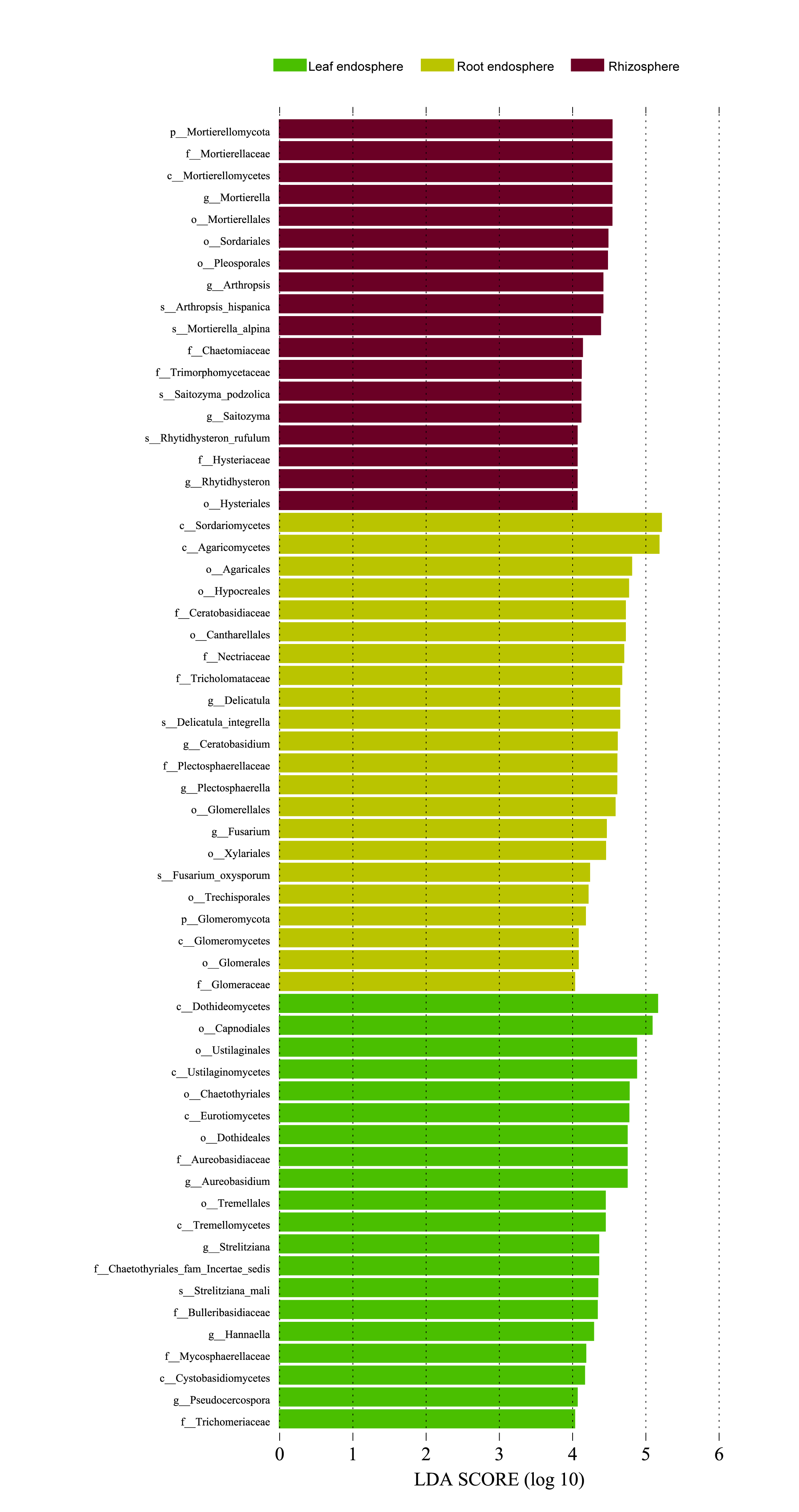

Supplement: FIGURE S5 — Histogram of the LDA scores computed for differentially abundant fungal taxa for each plant compartment. [file Image_5.TIF]

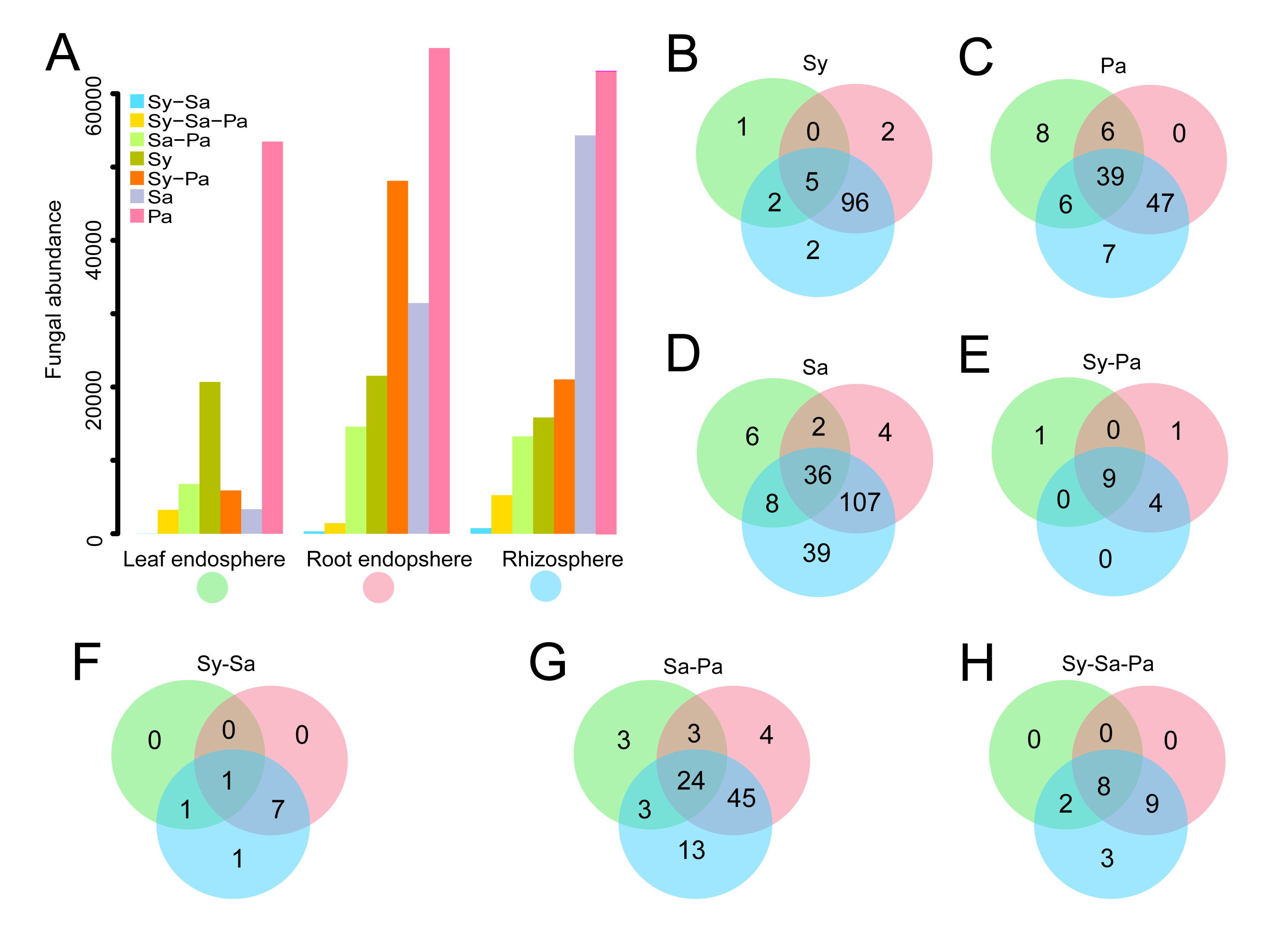

Supplement: FIGURE S6 — Trophic guilds of fungal communities in the three plant compartments. The trophic guilds were assigned through the FUNGuild data base. (B–H) Venn diagrams of the seven trophic guilds among compartments. Green, pink, and blue circles represent leaf endosphere, root endosphere and rhizosphere, respectively. Sy, symbiotroph; Pa, pathotroph; Sa, saprotroph; Sy-Pa, symbiotroph-pathotroph; Sy-Sa, symbiotroph-saprotroph; Sa-Pa, saprotroph- pathotroph; Sy-Sa-Pa, symbiotroph-saprotroph-pathotroph. [file Image_6.TIF]
