# Supplementary material for: Leaf and Root Endospheres Harbor Lower Fungal Diversity and Less Complex Fungal Co-occurrence Patterns Than Rhizosphere
Source: Front Microbiol. 2019 May 8;10:1015. doi: 10.3389/fmicb.2019.01015 (PMC6521803; doi:10.3389/fmicb.2019.01015)
Supplement: TABLE S1 — The 10 most abundant genera for the three different plant compartments. [file Table_1.DOCX]

**TABLE S1** The 10 most abundant genera for the three different plant compartments.

| Leaf endosphere | |  | Root endosphere | |  | Rhizosphere soil | |
| --- | --- | --- | --- | --- | --- | --- | --- |
| Genus | Reads |  | Genus | Reads |  | Genus | Reads |
| *Aureobasidium* | 48588 |  | *Delicatula* | 40928 |  | *Mortierella* | 32478 |
| *Strelitziana* | 22204 |  | *Plectosphaerella* | 36121 |  | *Arthropsis* | 18646 |
| *Hannaella* | 15379 |  | *Ceratobasidium* | 34880 |  | *Plectosphaerella* | 15174 |
| *Pseudocercospora* | 11736 |  | *Fusarium* | 25657 |  | *Delicatula* | 13971 |
| *Strigula* | 9605 |  | *Thanatephorus* | 10939 |  | *Fusarium* | 12156 |
| *Erythrobasidium* | 8713 |  | *Ilyonectria* | 7510 |  | *Rhytidhysteron* | 12152 |
| *Cladosporium* | 6721 |  | *Setophoma* | 7438 |  | *Saitozyma* | 10926 |
| *Pseudozyma* | 6583 |  | *Strigula* | 7129 |  | *Ceratobasidium* | 9436 |
| *Colletotrichum* | 5597 |  | *Metacordyceps* | 6380 |  | *Strigula* | 6933 |
| *Camptophora* | 4343 |  | *Psathyrella* | 5805 |  | *Staphylotrichum* | 6904 |
